# The prevalence of cognitive impairment in older Indian persons with cancer and brain metastases

**DOI:** 10.3332/ecancer.2022.1372

**Published:** 2022-04-07

**Authors:** Aditya Dhanawat, Vanita Noronha, Anant Ramaswamy, Shreya Gattani, Renita Castelino, Ratan Dhekale, Sarika Mahajan, Vijay Patil, Nandini Menon, Anuradha Daptardar, Vikram Gota, Shripad Banavali, Rajendra Badwe, Kumar Prabhash

**Affiliations:** 1 Department of Medical Oncology, Tata Memorial Centre, Homi Bhabha National Institute, Mumbai, Maharashtra 400012, India; 2 Department of Clinical Pharmacology, Tata Memorial Centre, Homi Bhabha National Institute, Mumbai, Maharashtra 400012, India; 3 Utsaah Foundation, Parel, Mumbai, Maharashtra 400012, India; 4 Department of Physiotherapy, Tata Memorial Centre, Homi Bhabha National Institute, Mumbai, Maharashtra 400012, India; 5 Department of Surgical Oncology, Tata Memorial Centre, Homi Bhabha National Institute, Mumbai, Maharashtra 400012, India

**Keywords:** neurocognitive decline, geriatric, brain metastases, cancer

## Abstract

**Background:**

Older patients with cancer are more vulnerable to the effects of cognitive impairment affecting their functional status, quality of life, compliance to treatment and ultimately survival. Cancer-related cognitive impairment may be due to the cancer itself or due to the treatment of cancer. There are no data regarding the prevalence of cognitive impairment in older persons with cancer and brain metastasis.

**Methods:**

This retrospective analysis was conducted on a prospectively collected data set of patients who attended the geriatric oncology clinic at a tertiary care comprehensive cancer centre in India from June 2018 to July 2021. Patients aged 60 years and above with malignancy were included. Cognition was assessed with the mini-mental status examination (MMSE); the Hindi MMSE was used for illiterate patients. A score of ≤23 on the MMSE was considered abnormal. Correlation between the presence of cognitive impairment and brain metastasis was tested using the chi-square test.

**Results:**

A total of 597 patients were included, of which 462 (77.4%) were male. The median age was 69 years (range: 60–100 years). All patients had solid tumours; 244 (40.9%) had lung, 189 (31.7%) had gastrointestinal and 75 (12.6%) had head and neck malignancies. Forty-one (6.9%) patients had brain metastases, of which 10 (24.4%) had solitary, 30 (73.2%) had multiple lesions and 1 (2.4%) had leptomeningeal metastases. Cognitive impairment was noted in 11 (26.8%) of the 41 patients with brain metastases and 91 (16.4%) of the 556 patients without brain metastases. There was no significant correlation between the presence of brain metastases and cognitive impairment, *p* = 0.086.

**Conclusion:**

Older persons with cancer and brain metastases were not found to have a higher occurrence of cognitive impairment than those without brain metastases in this study. The next step is to understand whether older persons with brain metastases are at a higher risk for cognitive decline as a result of therapeutic interventions such as cranial radiotherapy and chemotherapy.

## Background

There is a considerable increase in the population of older adults as a result of increased longevity due to better and wider healthcare facilities. According to the World Population Prospects 2019, by 2050, 1 in 6 people in the world (16%) will be above the age of 65, up from 1 in 11 (9%) in 2019. In India, this figure is projected to rise from 6.4% in 2019 to 8.6% by 2030 [1]. Aging is a strong risk factor for neurocognitive disorders such as Alzheimer’s disease (AD) and other dementias. According to a US population-based study, it is estimated that by the year 2050, the incidence of AD in persons older than 85 years would quadruple and between 75 and 85 years would double owing to the increase in life expectancy [2].

With advancements in screening and early diagnosis of cancer as well as improvements in cancer care, there has been an increase in the number of cancer survivors over the last decade and, consequently, there is a higher proportion of older adults with cancer as well. Almost 64% of the cancer survivors are aged 65 years or older [3].

Brain metastases are the most common intracranial malignancy in adults, affecting 20%–40% of all cancer patients. Primary cancers such as lung, breast and melanoma are most likely to metastasise to the brain [4]. The most common location of brain metastases is the cerebral hemispheres (70%), followed by the cerebellum (15%) and brainstem (<5%) [5, 6]. They are 10 times more likely to cause neurological impairment as compared to primary brain tumours [7]. Neurocognitive impairment is exhibited in nearly 90% of the persons with brain metastasis and the degree of dysfunction correlates more with the volume than the number of lesions [8]. Cancer-related cognitive changes and impairment might be due to the cancer itself. Additionally, cancer chemotherapy, radiation therapy and endocrine therapy may also cause cognitive impairment. Older patients with cancer may inherently be more vulnerable to the effects of cognitive impairment hampering their functional status, memory, attention, compliance to treatment and ultimately survival [9, 10]. Identification of cognitive impairment in older patients with cancer would enable us to curate personalised treatment options so as to prevent treatment induced cognitive impairment and also lay our focus on improvement in the quality of life in these patients.

Presently, there are no data on the prevalence of cognitive impairment in older patients with cancer and brain metastases. We, therefore, aimed to study the cognitive functioning in our older patients with cancer.

## Materials and methods

### Study design

This was a retrospective analysis of the data prospectively collected as part of the observational study on the cohort of patients who attended the geriatric oncology clinic at a tertiary care comprehensive cancer centre in India from June 2018 to July 2021. The study was approved by the Institutional Ethics Committee. Written informed consent was obtained from the patients prior to evaluation in the geriatric oncology clinic during the prospective portion of the study; the IEC granted a waiver for the requirement of obtaining written informed consent for the patients who were retrospectively enrolled in the study. The study was conducted according to the ethical guidelines outlined in the Declaration of Helsinki, Good Clinical Practice guidelines and the Indian Council of Medical Research. The study was registered with the Clinical Trials Registry India (CTRI/2020/04/024675).

### Study participants

Patients aged 60 years and above with a diagnosis of malignancy, who were evaluated in the geriatric oncology clinic and had undergone a geriatric assessment were included. Patients or caregivers who refused to undergo the geriatric assessment (GA); patients who were completely disabled (Eastern Cooperative Oncology Group (ECOG), performance status (PS) 4); and patients who could not undergo mini-mental status examination (MMSE) were excluded from the study.

### End points

The primary end point of this study was to determine the prevalence of cognitive impairment in older patients with cancer that was metastatic in the brain. Our secondary end point was to determine whether there was any correlation between the presence of cognitive impairment and the presence of brain metastases in older patients with cancer.

### Study methodology

Details regarding the socio-demographic information, primary tumour location, baseline radiological scans and the presence of brain metastases were documented at the time of the GA. The geriatric assessment in our setting took place at the treatment planning phase and prior to initiation of chemotherapy, radiation therapy or surgery. All investigations and management were performed by the treating physicians. Patients who had neurological symptoms underwent radiologic brain imaging. As a general departmental policy, radiological imaging of the brain in the form of a magnetic resonance imaging (MRI) scan was not routinely done for patients without neurological symptoms in the palliative setting. Brain metastases were also diagnosed in asymptomatic patients who underwent a whole-body 18-fluorodeoxyglucose positron emission tomography/computed tomography (PET-CT) as a part of the staging work-up. Cognition was assessed routinely in all patients as a part of the GA. Cognition was assessed with the MMSE [11]; the Hindi MMSE was used for illiterate patients [12]. A score of 18–23 on the MMSE was considered as mild cognitive impairment and a score ≤17 was considered severe cognitive impairment.

### Statistical analysis

As this was a retrospective study, no formal sample size calculation was performed. We included all patients who fulfilled the study eligibility criteria in the time period of the study. Data analysis was performed in the Statistical Program for the Social Sciences (IBM Corp. Released 2017. IBM SPSS Statistics for Windows, Version 25.0. Armonk, NY: IBM Corp.). Descriptive statistics were presented using absolute numbers and simple percentages. Quantitative data were presented using median and interquartile range. Correlation between the presence of cognitive impairment and brain metastases was tested using the chi-square test (Fisher’s exact test).

## Results

A total of 616 patients attended the geriatric oncology clinic between June 2018 and July 2021, of which 19 (3.1%) patients were not tested for cognition owing to inability to complete the MMSE. Thus, 597 patients were included in the study. The median age of presentation was 69 years (range: 60–100 years) (Table 1).

MRI of the brain was done in 117 (19.6%) patients and PET-CT in 287 (48.1%) patients. In the remaining 193 (32.3%) patients, no brain imaging was performed.

Overall, 41 (6.9%) patients had brain metastases, of which 26 (63.4%) patients were symptomatic for brain metastases. Symptoms included sensory/motor weakness (13, 31.7%), altered sensorium (11, 26.8%), headache (9, 21.9%), seizures (3, 7.3%) and cerebellar symptoms (3, 7.3%). Twenty-two (53.6%) patients were on steroids (dexamethasone) and 12 (29.3%) on an anti-epileptic medication (levetiracetam). Thirty-two (78.0%) patients underwent whole-brain radiotherapy, one (2.4%) underwent stereotactic radiosurgery and one (2.4%) patient underwent surgical metastatectomy of the brain lesion.

Of these 41 patients with brain metastases, 38 (92.7%) were diagnosed based on MRI and the remaining 3 (7.3%) patients on PET-CT. Ten patients (24.4%) had solitary, 30 (73.2%) had multiple lesions and 1 (2.4%) had leptomeningeal metastases. Thirty-seven (90.2%) patients had cerebral metastases, 3 (7.3%) had cerebellar metastases and 1 (2.5%) had leptomeningeal metastasis. Brain metastases were present mostly in lung cancer (39 patients, 95.1%), followed by genito-urinary cancer (1 patient, 2.4%) and head and neck cancer (1 patient, 2.4%).

The median MMSE score was 28 (interquartile range (IQR): 25–29). The median MMSE in patients with brain metastases was 26 (IQR: 22–28) and without brain metastases was 28 (IQR: 25–29).

The median MMSE in patients with solitary metastasis was 27 (IQR: 20–29.75), whereas the median MMSE in patient with multiple metastases was 26 (IQR: 24–28). The MMSE in patient with leptomeningeal metastasis was 29. The median MMSE in patients with cerebral metastases was 26 (IQR: 20–28) and the median MMSE in patients with cerebellar metastases was 26 (IQR: 26–27).

**Figure 1.**
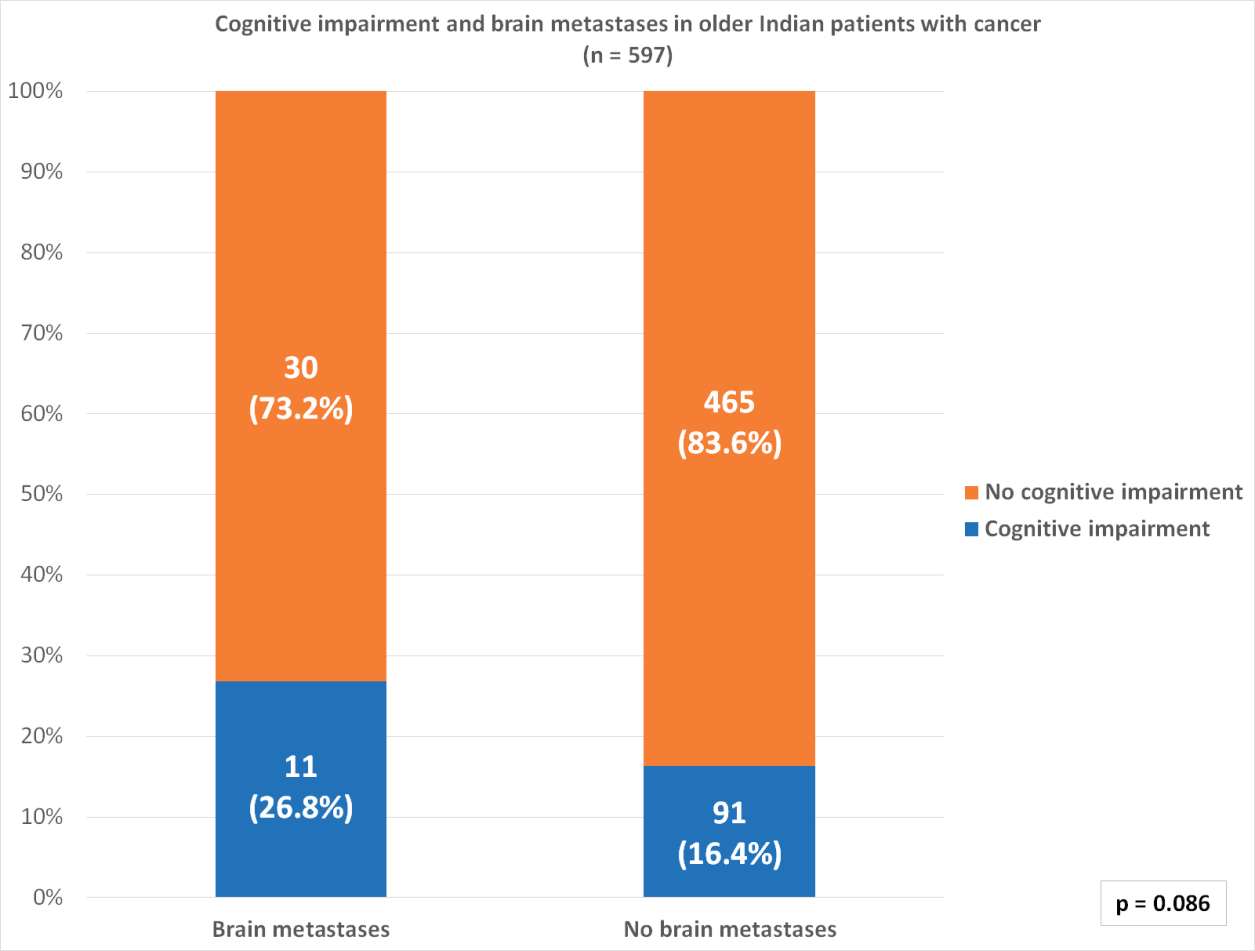
Correlation between cognitive impairment and brain metastases in older Indian patients with cancer.

Of the entire cohort, 102 (17.1%) patients had cognitive impairment and 25 (4.2%) patients had severe cognitive impairment. Cognitive impairment was noted in 11 (26.8%) of the 41 patients with brain metastases and 91 (16.4%) out of 556 patients without brain metastases (Figure 1). Severe cognitive impairment was present in 5 patients (12.2%) with brain metastasis and 20 (3.6%) without brain metastasis. There was no statistically significant correlation between the presence of brain metastases and cognitive impairment (*p* = 0.086).

## Discussion

We found that 6.9% of older patients with cancer had brain metastases and 17.1% of them had cognitive impairment. Historically, the overall prevalence of brain metastases has been found to be higher in autopsy studies than population-based studies. Autopsy studies [13, 14] have reported the incidence of brain metastases to be between 9% and 26%, but these studies were conducted over 40 years ago and nowadays, autopsies are rarely done. Some of the population-based studies in the early 2000s [15, 16] have reported the prevalence to be in the range of 8.5%–9.6% among all cancer patients, irrespective of age. In a recent SEER-Medicare study [17], the incidence proportions of synchronous brain metastases were in the range of 1.8%–9.6%. However, this study only considered patients with lung, breast and skin cancer and did not comment on incidence proportions in older patients. It is imperative to note that the incidence of brain metastases would vary widely depending on primary tumour location and histology. In another recent study by Singh *et al* [18], the incidence of brain metastases across all primary sites and age groups ranged roughly from 7.1 to 7.4 persons per 1,00,000 population.

The prevalence of cognitive impairment in the older population of South India has been reported to be 8.4% [19]. Other Indian studies have reported the prevalence of cognitive impairment in the older population to be in the range of 14%–26% [20–22]. However, these studies were conducted on a general older population and not on older patients with cancer. The SEER-Medicare studies [23–25] reported the estimated prevalence of dementia in older (age 65 years or more) patients with cancer to be 3.8%–7%. This difference could be attributed to racial variations. Besides, these studies were conducted on patients with breast, colon and prostate cancer. It is unknown whether there is a difference in the prevalence of cognitive impairment based on the primary tumour location. In a review by Janelsins *et al* [26], cancer-related cognitive impairment in patients of all age groups was reported to be around 30% at baseline, 75% during treatment and 35% following the completion of treatment. However, Mandelblatt *et al* [27] found no statistical difference in cognitive impairment between older patients with non-metastatic breast cancer (14%) and matched community controls (15%). In the ONCODAGE study, 20% of the 1,435 patients with cancer enrolled had an abnormal MMSE [28]. In the systematic review of 35 studies by Hamaker *et al* [29] on the effect of the results of the geriatric assessment on the treatment decisions and outcomes in older persons with cancer, recommendations for cognitive-based therapy and delirium prevention were made in 5%–35% (mean = 14.9%) of the patients [29]. Cognitive impairment was noted in 17.3% of our cohort of older patients with cancer, which is similar to what has been described in the above studies.

The interplay between aging, cognition and brain metastasis is not well documented in the literature. However, it was expected that older patients with brain metastases may have a higher frequency of cognitive impairment due to the involvement of the brain parenchyma, depression/distress and the use of supportive medications like corticosteroids or anti-epileptics (e.g., carbamazepine, phenytoin etc.) [30]; we did not find any statistical increase in cognitive impairment in the presence of brain metastases.

Patients with brain metastases have neurocognitive dysfunction in various areas including impairments in verbal memory, attention, executive functioning and language in relation to healthy controls based on a study in USA which undertook neuropsychological evaluation of all participants [31]. These neurocognitive deficits were noted in patients who had minimal functional impairment and were able to carry out their activities of daily living (ADL). There occurs a reduction in the volume of the white and grey matter of patients between 1 and 20 years after the completion of chemotherapy because of the action of chemotherapeutic agents on neural progenitor cells, mature post-mitotic oligodendrocytes, pro-oxidative effects, impaired hippocampal neurogenesis, toxic neurotransmitter release and disruption of blood vessels density. Endocrine therapy, like selective oestrogen receptor modulator and aromatase inhibitors in breast cancer, targeted agents like vascular endothelial growth factor (VEGF) inhibitors (Sunitinib and Bevacizumab) and lenalidomide has been known to cause cognitive impairment [32].

Patients with brain metastases may experience neurologic symptoms, sleep disturbances, mood disturbances, pain and fatigue. Mental and physical fatigue can also affect cognitive functioning. Strong correlation has been observed between neurocognitive impairment and patient-rated quality of life (QOL), such as Functional Assessment of Cancer Therapy-Brain (FACT-Br) ratings. A thorough assessment of patient QOL is needed, and not just the performance status. Pharmacologic interventions (anti-depressants and mood stabilisers) and psycho-social interventions (psychotherapy and support groups) may help to minimise the adverse cognitive effects of the disease and improve patient QOL regardless of the stage of the illness. Cognitive function needs to be preserved as long as possible [33].

## Limitations

There are certain limitations to our study and future directions should be considered. First, participants were included from a single centre and our sample size was small. Only a small proportion of patients (6.9%) had brain metastasis, thereby limiting the ability to universalise our study findings to all older persons with brain metastases. Second, there was 32.3% of the patients who did not undergo brain imaging or PET-CT. Some proportions of these patients may have had asymptomatic brain metastases and remained undiagnosed. Third, patients in this study had heterogeneous primary cancers and varied presentations of brain metastasis in terms of size and location. Fourth, we assessed cognitive impairment based on MMSE and did not perform comprehensive neuropsychological testing. The MMSE is influenced by non-cognitive domains and is confounded by language, levels of literacy and cultural and ethical norms. However, we did use the Hindi MMSE for patients who were illiterate. MMSE is not very useful in detecting mild cognitive impairment or recording prognosis in severe dementia. MMSE is associated with a ceiling effect in well-educated persons leading to false negatives. MMSE does not assess domain-specific impairments such as executive function, neglect, apraxia and aphasia [34]. There is a need to stratify and investigate the differences in cognition based on these characteristics.

Future research to assess the presence of cognitive impairment in older patients with brain metastases would help in determining management strategies like enhanced social support, closer supervision and cognitive behavioural therapy. It would be important to perform a longitudinal evaluation of cognitive functioning in patients with brain metastases to determine the effects of various therapeutic interventions. Finally, more studies in this direction with larger and multi-institutional cohort of older persons with brain metastasis are needed to cross-validate these results.

## Conclusion

Older persons with cancer and brain metastases were not found to have a higher incidence of cognitive impairment than those without brain metastases in this study. The next step is to understand whether these patients are more at risk for cognitive decline due to therapeutic interventions like cranial radiotherapy and chemotherapy.

## Conflicts of interest

None.

## Funding

This research did not receive any specific grant from funding agencies in the public, commercial or not-for-profit sectors.

## Figures and Tables

**Table 1.** Clinico-demographic details of patients who attended the geriatric oncology clinic.

Sl. No.	Patient characteristics	No. of patients (*N* = 597)
1	Age (in years)	
	60–69	300 (50.2%)
	70–79	256 (42.9%)
	80–89	38 (6.4%)
	>90	3 (0.5%)
2	Sex	
	Females	135 (22.6%)
	Males	462 (77.4%)
3	Education level	
	Did not attend school	89 (14.9%)
	Primary school	61 (10.2%)
	Secondary school	254 (42.6%)
	Undergraduation	73 (12.2%)
	Graduation	80 (13.4%)
	Postgraduation	40 (6.7%)
4	Primary tumour location	
	Lung	244 (40.9%)
	Gastro-intestinal	189 (31.7%)
	Head and Neck	75 (12.6%)
	Genito-urinary	68 (11.4%)
	Breast	9 (1.5%)
	Unknown primary	12 (2.0%)
5	Stage of the disease	
	I	3 (0.5%)
	II	51 (8.5%)
	III	210 (35.2%)
	IV	333 (55.8%)
6	Intent of treatment	
	Palliative	348 (58.3%)
	Curative	249 (41.7%)
7	ECOG PS	
	0	46 (7.7%)
	1	312 (52.3%)
	2	167 (28.0%)
	3	72 (12.1%)

ECOG, Eastern Cooperative Oncology Group; PS, Performance Status
